# Probing Chiral Recognition on Amylose Tris(3,5‐Dimethylphenylcarbamate) Using Cinnamyl 2‐Aminoanilides: The Subtle Impact of Aliphatic Substituents

**DOI:** 10.1002/elps.70109

**Published:** 2026-05-19

**Authors:** Selen Gözde Kaya, Alessia Raucci, Clemens Zwergel, Antonello Mai, Sergio Valente, Roberto Cirilli

**Affiliations:** ^1^ Dipartimento di Chimica e Tecnologie del Farmaco Università degli Studi di Roma “La Sapienza” P.le A. Moro 5 Rome Italy; ^2^ Faculty of Pharmacy Gazi University Department of Pharmaceutical Chemistry Ankara Türkiye; ^3^ Centro Nazionale per il Controllo e la Valutazione dei Farmaci Istituto Superiore di Sanità Viale Regina Elena 299 Rome Italy

## Abstract

This study investigates the chiral recognition behavior of the polysaccharide‑based chiral stationary phase amylose tris(3,5‑dimethylphenylcarbamate) (Chiralpak AD‑3) under polar organic conditions, using a series of racemic cinnamyl 2‑aminoanilides differing only in the nature of their aliphatic substituents. Remarkably large differences in enantioselectivity were observed among closely related compounds, with enantioseparation factors reaching exceptionally high values (α up to 37) when neat 2‑propanol was employed as the mobile phase. In contrast, replacement of 2‑propanol with 1‑propanol led to a dramatic reduction or complete loss of chiral discrimination, highlighting the crucial role of solvent structure in modulating analyte–selector interactions. Analysis of retention and selectivity trends suggests that chiral recognition primarily arises from hydrogen bonding involving the anilide NH_2_ group, combined with other interactions modulated by the aliphatic side chains. These findings emphasize how subtle structural modifications, together with mobile phase composition, can profoundly affect enantioselectivity on polysaccharide‑based chiral stationary phases.

## Graphical abstract

This study investigates how subtle aliphatic substituent differences in racemic cinnamyl 2‐aminoanilides affect chiral recognition on amylose tris(3,5‐dimethylphenylcarbamate). Pronounced enantioselectivity changes arose from minimal structural variations. Mobile phase effects were examined, with 2‐propanol improving resolution. Results suggest contributions from hydrogen bonding and steric, dispersive, and solvophobic interactions.

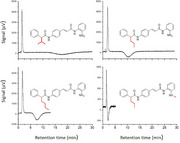

## 1 Introduction

Enantioselective separation of chiral compounds is a key objective in modern analytical chemistry with critical applications in the pharmaceutical, agrochemical, and fragrance industries. Among the available techniques, high‐performance liquid chromatography (HPLC) on chiral stationary phases (CSPs) is one of the most effective and commonly used methods for separating enantiomers at both the analytical and preparative levels.

Polysaccharide‐based CSPs, especially those derived from amylose and cellulose carbamates, have received significant attention because of their broad enantioselectivity and versatility [1]. Amylose tris(3,5‐dimethylphenylcarbamate), a chiral selector found in many commercial CSPs, shows outstanding resolving ability under normal‐phase and polar organic conditions [1, 2, 3, 4, 5, 6].

The enantioselective recognition mechanism of the coated‐type Chiralpak AD‐3 CSP, which is based on amylose tris(3,5‐dimethylphenylcarbamate), results from a finely balanced network of noncovalent interactions between the chiral selector and the analyte. The general principles governing chiral recognition on polysaccharide‑based CSPs have been extensively investigated and comprehensively reviewed, highlighting the complex and cooperative nature of these interactions [7]. The carbamate groups have dual interaction abilities: the NH groups serve as hydrogen‐bond donors, and the carbonyl oxygens act as hydrogen‐bond acceptors [2, 5, 6]. This allows for directional and stereoselective hydrogen bonding with complementary functional groups on the analyte.

This moiety helps create a chiral environment that is highly sensitive to even minor structural differences, enabling it to efficiently discriminate between enantiomers. Furthermore, the aromatic rings of the carbamate substituents support π–π stacking and dispersive interactions, which contribute to different stabilization. The methyl groups at the 3‐ and 5‐positions of the phenyl rings play a dual role, modulating both steric hindrance and the electronic properties of the NH groups, thus fine‐tuning the hydrogen bonding ability of the selector [7].

**FIGURE 1:**
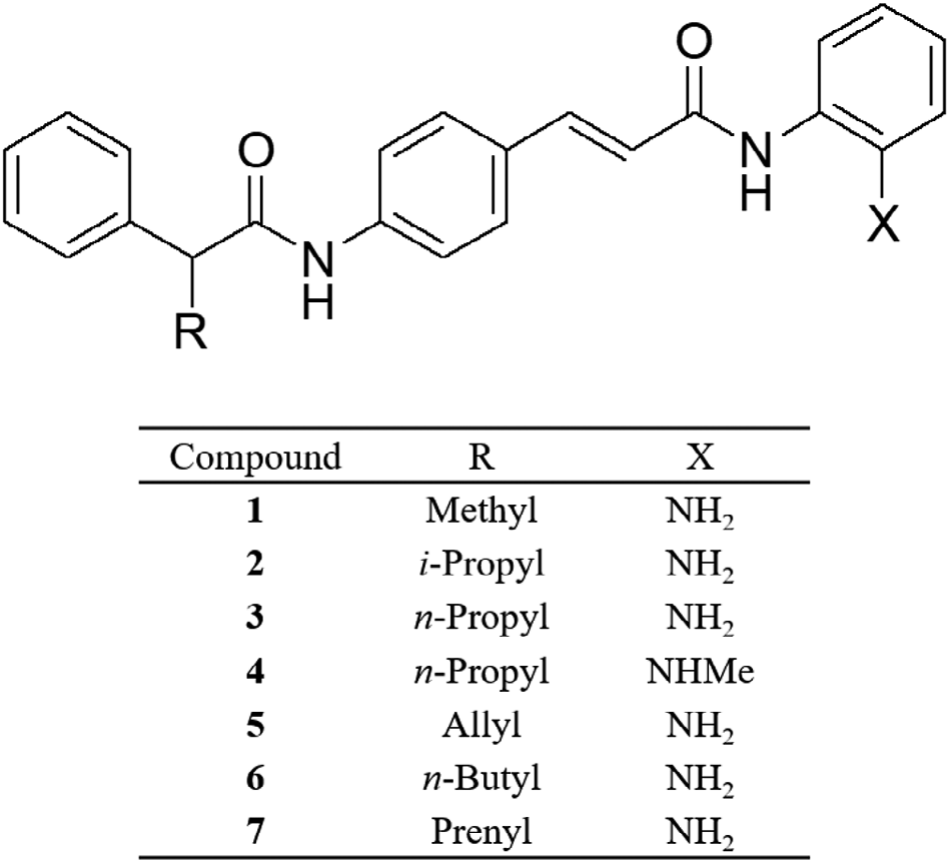
Structures of **1**–**7**.

Importantly, the ability of polysaccharide‐based CSPs to recognize chiral compounds is influenced not only by the structural features of the selector and the selectand, but also by the composition of the mobile phase [1, 6, 8, 9, 10, 11]. Numerous studies have demonstrated that the mobile phase plays an active role by modulating both analyte solvation and the conformational ensemble accessible to the chiral selector, an effect that is particularly pronounced under polar organic conditions [12]. Even subtle variations in mobile phase composition can markedly influence the strength and nature of analyte–selector interactions through changes in solvent polarity, proticity, and hydrogen‐bonding ability, with direct consequences on enantioselectivity. In this context, 2‐propanol has emerged as an effective solvent for improving chiral discrimination on Chiralpak AD‐3, whether used as a pure mobile phase or as a component of binary solvent systems [13, 14, 15, 16, 17, 18, 19]. To study the effect of subtle structural variations in the analyte on chiral recognition under these conditions, we selected a series of cinnamyl 2‐aminoanilide derivatives as chiral probes (Figure 1). These molecules have multiple functional groups that can engage in hydrogen bonding, π–π interactions, and dispersive forces with the chiral selector.

Recent studies have shown that enantioselectivity in multifunctional systems often arises from a complex interplay of weak, cooperative interactions involving different motifs of the chiral analyte and cannot always be attributed solely to identifiable hydrogen‑bonding patterns [20]. As demonstrated by our group in a previous study, the nature of the aliphatic substituent at the stereogenic center plays a decisive role in modulating enantioselectivity in cinnamyl 2‑aminoanilide derivatives [19]. For instance, the use of pure 2‑propanol as the mobile phase at 25°C resulted in an enantioseparation factor (*α*) of 9.20 for the methyl‑substituted compound **1** (Figure 1). Replacing the methyl group with an isopropyl substituent (compound **2**) dramatically increased α to 45.59. Conversely, replacing 2‑propanol with ethanol resulted in a significant decrease in enantioselectivity (*α* = 2.16 and 5.37 for compounds **1** and **2**, respectively), underscoring the critical influence of mobile phase composition on chiral recognition. Based on these observations, the present study expands the original series of cinnamyl 2‐aminoanilides by introducing additional substituents at the stereogenic center. These include the n‐propyl (compound **3**), n‐butyl (compound **5**), allyl (compound **6**), and prenyl (compound **7**) groups. To further probe the role of hydrogen bonding in chiral recognition, a methyl group was introduced at the NH_2_ position of the anilide moiety in compound **2**, yielding compound **4**.

## 2 Experimental

### 2.1 Materials, Instruments, and Chromatographic Conditions

Analytical HPLC analysis of compounds **1**–**7** was performed using a commercially available Chiralpak AD‐3 column (100 mm × 4.6 mm i.d., 3 µm particle size; Chiral Technologies Europe, Illkirch, France). HPLC‐grade solvents were sourced from Sigma‐Aldrich (St. Louis, MO, USA). The HPLC system consisted of a PerkinElmer 200 LC pump (Norwalk, CT, USA) with a Rheodyne manual injector (Cotati, CA, USA), a 50 µL sample loop, a Jasco UV/CD‐2095 Plus detector (Tokyo, Japan), and a PerkinElmer LC 101 column thermostat (Sunnyvale, CA, USA). Data acquisition and processing were conducted using Clarity software (DataApex, Prague, Czech Republic).

Standard solutions were prepared by dissolving approximately 1 mg of each compound in 5 mL of ethanol. The injection volume was set to 10 µL. The hold‐up time was calculated at 40°C and a flow rate of 1 mL min^−1^ by using 1,3,5‐tri‐tert‐butylbenzene as the nonretained marker and pure 2‐propanol as the mobile phase.

The electronic circular dichroism (ECD) spectra were recorded in a 0.1 cm path length quartz cell at 25°C using a Jasco (Tokyo, Japan) model J‐700 spectropolarimeter. The intensities of spectra are expressed as ellipticity values (mdeg).

### 2.2 Synthesis of 3–7

Based on our previous work [21], the title compounds were prepared using an optimized procedure involving the key intermediate ethyl 4‐aminocinnamate (see the Supporting Materials for experimental details and product characterization).

## 3 Results and Discussion

### 3.1 Enantioselective HPLC

The enantioseparation of compounds **1** and **2** was initially evaluated under analytical conditions using a Chiralpak AD‐3 column (100 mm × 4.6 mm, 3 µm) at 40°C, with neat 2‐propanol as the mobile phase. The retention factors (*k_1_*) of the first‐eluting enantiomers and the corresponding enantioseparation factors (*α*) are summarized in Table 1. As previously observed at 25°C [19], compound **2** exhibited exceptional enantioselectivity (*α* = 37.06), while its methyl‐substituted analogue **1** showed a markedly lower value (*α* = 6.18).

**TABLE 1:** Retention factors (k_1_) for the first eluting enantiomer and enantioseparation factors (*α*) of compounds **1**–**7**.

Compound	Mobile phase	$k_{1}$	*α*
**1**	2‐Propanol	1.26	6.18
	1‐Propanol	0.48	1.84
**2**	2‐Propanol	0.45	37.06
	1‐Propanol	0.35	3.89
**3**	2‐Propanol	0.90	10.29
	1‐Propanol	0.45	2.04
**4**	2‐Propanol	0.44	2.53
	1‐Propanol	0.23	1.00
**5**	2‐Propanol	0.76	9.88
	1‐Propanol	0.45	1.91
**6**	2‐Propanol	0.66	9.98
	1‐Propanol	0.45	1.95
**7**	2‐Propanol	0.42	15.77
	1‐Propanol	0.43	1.63

*Note*: Chromatographic conditions: Column, Chiralpak AD‐3 (100 mm × 4.6 mm, 3 µm); flow rate, 1.0 mL min^−1^; temperature, 40°C; detection, UV/CD at 320 nm.

These values represent the free‐energy differences between the corresponding diastereomeric complexes (ΔΔ*G*° = −RT ln α, where *R* is the gas constant and *T* the absolute temperature), amounting to −2.25 and −1.13 kcal mol^−^^1^ for **2** and **1**, respectively. These results indicate a stronger interaction of the second‐eluting enantiomer with the CSP.

When the mobile phase was changed to 1‐propanol, there was a sharp decrease in enantioselectivity for both analytes (*α* = 1.84 and 3.89; ΔΔ*G*° = −0.38 and −0.84 kcal mol^−1^ for **1** and **2**, respectively).

This sharp decline in enantioselectivity emphasizes the significant role of solvent structure in chiral recognition. The branched structure of 2‐propanol likely interacts differently with the chiral stationary phase compared with the linear 1‐propanol, influencing the solvation environment and possibly changing the accessibility or conformation of the chiral cavities in the selector. These solvent‐driven changes may weaken essential noncovalent interactions, such as hydrogen bonds and dispersive forces, between the enantiomers and the CSP, ultimately reducing the efficiency of chiral discrimination.

Comparing the retention and enantioselectivity factors of compound **2** with those of its structural analogue **3** (Figure 1), which was obtained by replacing the isopropyl group with a *n*‐propyl moiety, further underscores the critical influence of the alkyl substituent on enantioselectivity.

Under chromatographic conditions using 2‐propanol, the substitution caused a significant decrease in the enantioseparation factor from 37.06 (compound **2**) to 10.29 (compound **3**), corresponding to a ΔΔ*G*° drop from −2.25 to −1.36 kcal mol^−1^. When 1‐propanol was used as the mobile phase, the reduction in enantioselectivity was less pronounced, with α values decreasing from 3.89 to 2.04 and the corresponding ΔΔ*G*° values from −0.84 to −0.45 kcal mol^−1^.

As shown in Table 1, the insertion of aliphatic groups such as allyl (compound **5**), *n*‐butyl (compound **6**), and prenyl (compound **7**) resulted in enantioseparation factors ranging from about 10 to 16, with the highest value observed for compound **7** when 2‐propanol was used as the mobile phase. As expected, all compounds exhibited significantly lower enantioselectivity in 1‐propanol.

**FIGURE 2:**
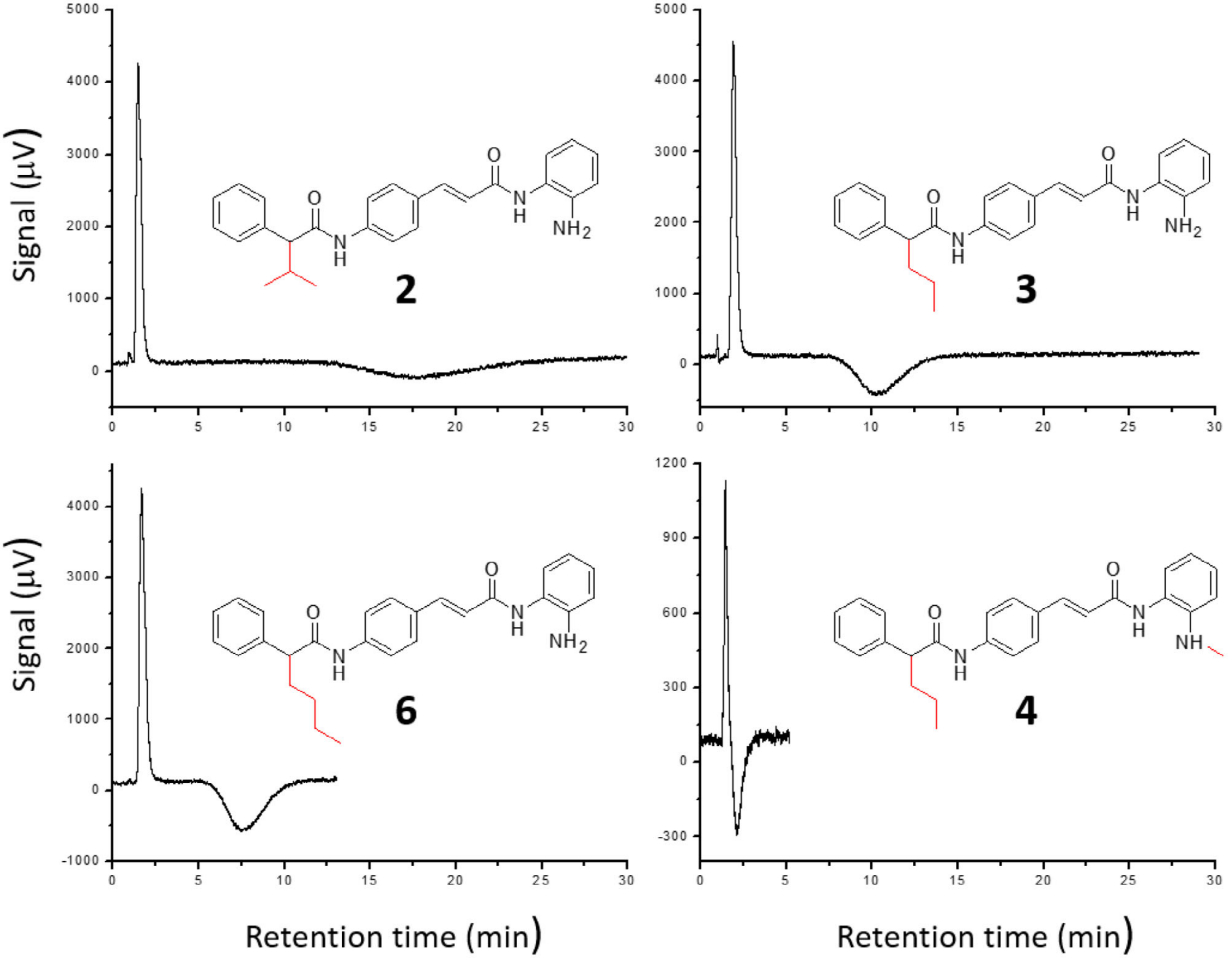
Typical chromatograms illustrating the differences in enantioseparation of **2**, **3**, **4**, and **6** using pure 2‐propanol as a mobile phase. Chromatographic conditions: Column, Chiralpak AD‐3 (100 mm × 4.6 mm, 3 µm); column temperature, 40°C; flow rate, 1.0 mL min^−1^; detection, CD at 320 nm.

The chromatograms for the enantioseparation of compounds **2**, **3**, **4**, and **6**, shown in Figure 2, demonstrate the distinctive response of the Chiralpak AD‐3 CSP to subtle structural modifications within the cinnamyl 2‐aminoanilide scaffold.

An inspection of the retention data reveals that the first‑eluting enantiomers exhibit very low affinity for the CSP in all cases, eluting close to the hold‑up time (*t*_0_ = 1.00 min). Consequently, the observed enantioselectivity is mainly governed by the interaction of the second‑eluting enantiomers with the Chiralpak AD‐3 CSP. The very broad and highly dispersed peaks are indicative of stronger and possibly heterogeneous interactions between the second‑eluting enantiomers and the CSP, resulting in slow mass‑transfer kinetics and pronounced peak broadening.

Two key factors emerged from the enantioselective HPLC analysis.

(i) Compounds bearing alkyl substituents of variable size at the stereogenic center exhibited pronounced solvent‑dependent enantioselectivity on the amylose‑based Chiralpak AD‑3 CSP. Both the size and branching of the aliphatic side chain influence steric, dispersive, and solvophobic interactions with the chiral selector.
(ii) The mobile‑phase composition strongly modulates these interactions, emphasizing the importance of fine structural tuning of the analytes to achieve high enantioselectivity on polysaccharide‑based CSPs. In particular, when 2‑propanol was used as the alcohol modifier, exceptionally high enantioseparation factors were observed (*α* values of up to 37), whereas replacing 2‑propanol with 1‑propanol resulted in a dramatic decrease in enantioselectivity.

Although only moderate differences in hydrogen‑bond donating ability are expected between these two alcohol isomers, extensive literature demonstrates that alcohol modifiers can profoundly affect the supramolecular organization of amylose tris(3,5‑dimethylphenylcarbamate) selectors, resulting in substantial changes in chiral recognition [6, 8, 9].

Solid‑state NMR studies by Wenslow et al. [9] revealed that alcohol modifiers play a role beyond simple elution, becoming incorporated into the polymeric selector and forming distinct solvent–CSP complexes whose structure depends strongly on the size and steric bulk of the alcohol. Specifically, 2‐propanol was shown to displace hexane more efficiently than primary alcohols and induce a more ordered, well‐defined amylose backbone conformation. In contrast, less hindered alcohols promote a more flexible, heterogeneous polymer structure.

Further structural insight has been obtained from NMR and molecular modeling studies. These studies revealed that amylose tris(3,5‐dimethylphenylcarbamate) adopts a helical secondary structure whose pitch and rigidity are highly sensitive to solvation effects [6, 9]. Therefore, changes in mobile‐phase composition can alter the geometry and accessibility of the chiral cavities responsible for enantioselectivity. These effects are nonlinear and can be dramatic, which explains why closely related solvents, such as 1‐ and 2‐propanol, can lead to dramatically different chromatographic outcomes.

From a mechanistic perspective, secondary alcohols, such as 2‐propanol, are bulkier and less effective at deeply penetrating nonenantiodiscriminating regions of the polymer. Consequently, they are believed to stabilize a more rigid, stereochemically defined selector conformation that favors multipoint binding interactions and shape‐selective recognition with analytes bearing sterically differentiated substituents. In contrast, 1‐propanol may solvate the carbamate groups more extensively due to its lower steric demand, increasing conformational averaging and weakening differential interactions between enantiomers.

These observations align with the findings of numerous chromatographic studies indicating that slight variations in the alcohol modifier in the mobile phase can significantly impact retention, enantioselectivity, and even elution order on amylose‐based CSPs [6, 7, 9, 22]. This effect is often attributed to solvent‐driven conformational rearrangements of the selector rather than direct analyte‐solvent interactions.

The last structural modification to the cinnamyl 2‐aminoanilide scaffold involved methylating the 2‐NH_2_ group, which can potentially form hydrogen bonds with complementary CONH functionalities on the Chiralpak AD‐3 CSP. Although the molecule still retains an amide group adjacent to the stereocenter, which can also participate in hydrogen bonding, the replacement of the NH_2_ hydrogen with a methyl group (compound **4**) caused a marked decrease in enantioselectivity: the enantioseparation factor fell from *α* = 10.29 (compound **3**) to *α* = 2.53. This was mainly due to about a 50% reduction in the retention of the second‐eluting enantiomer (*k*_2_ decreased from 11.43 to 4.63).

This chromatographic behavior corresponds to a reduction in the overall selector–selectand interaction free energy (ΔΔ*G*°) from −1.70 to −0.56 kcal mol^−^^1^, indicating a loss of approximately 1.14 kcal mol^−^^1^. This decrease likely stems from the removal of an N–H hydrogen‐bond donor, which would otherwise interact with the carbamate functionalities of the CSP. When 1‐propanol was employed as the mobile phase, enantioseparation was completely suppressed (*α* = 1), further emphasizing the crucial role of this hydrogen‐bond donor in facilitating chiral recognition under these conditions.

### 3.2 Absolute Configuration Assignment

ECD spectroscopy is a widely recognized and powerful technique for determining the absolute configuration of chiral molecules. Many studies have confirmed this method, which compares the experimental ECD spectra of enantiopure or enantioenriched compounds with reference data or theoretical predictions [23, 24, 25]. A key advantage of ECD is its high sensitivity: only small quantities of sample (typically 0.2–0.5 mg) are required, making it especially useful for compounds isolated by enantioselective HPLC, even on analytical‐scale columns.

**FIGURE 3:**
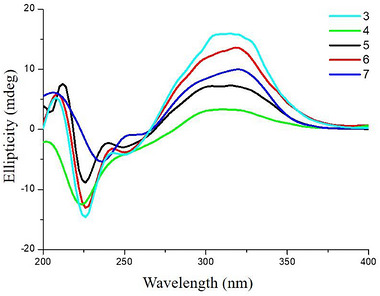
ECD spectra of the first eluted enantiomers of **3**–**7** recorded in 2‐propanol solution

In this study, the enantiomers of compounds **3**–**7** were isolated by HPLC on a Chiralpak AD‑3 column (100 mm × 4.6 mm) using 2‑propanol as the mobile phase, and their ECD spectra were recorded directly in solution. As shown in Figure 3, the ECD spectra of the first‑eluting enantiomers were only marginally affected by the nature of the alkyl substituent at the stereogenic center. All spectra closely resembled that of (*S*)‐**1** [19], displaying a broad, weak negative band in the 320–280 nm region, followed by a more intense negative ECD band at 225–230 nm and a positive one around 205 nm. The spectra of the second‑eluting enantiomers (not shown) were mirror images, confirming their enantiomeric relationship.

The strong similarity between the ECD profiles of compounds **3**–**7** and that of (*S*)‑**1** supports the assignment of the (*S*)‐configuration to the first‑eluting enantiomers, and consequently the (*R*)‐configuration to the more retained ones on the Chiralpak AD‑3 CSP. These results highlight the effectiveness of combining ECD spectroscopy with reliable chromatographic enantioseparation as a rapid, nondestructive, and sample‑efficient approach for establishing absolute configuration, particularly in studies aimed at elucidating structure–enantioselectivity relationships.

Interestingly, monitoring the online ECD signal at the diagnostic wavelength of 320 nm during HPLC enantioseparations revealed a consistent correlation between the ECD response and the absolute configuration of the analytes. As depicted in Figure 2, the first‐eluting enantiomers of all cinnamyl 2‐aminoanilide derivatives tested on the Chiralpak AD‐3 CSP displayed a positive ECD signal at 320 nm. This consistent CD response supports a reliable empirical assignment of absolute configuration, eliminating the need to collect enantiomerically enriched fractions of structurally related cinnamyl 2‐aminoanilides.

## 4 Conclusion

This study demonstrates that subtle aliphatic modifications in cinnamyl 2‐aminoanilide derivatives can cause significant and often unpredictable variations in enantioselectivity on the polysaccharide‐based Chiralpak AD‐3 CSP, with α values ranging from 6 to 37 at 40°C when using neat 2‐propanol as the mobile phase. The pronounced effect of the mobile phase, particularly the ability of 2‐propanol to enhance chiral resolution, underscores the active role of the mobile phase in modulating selector–selectand interactions.

The significant drop in enantioselectivity observed upon N‐methylation of the aromatic NH_2_ group of the anilide moiety points out the importance of hydrogen bonding in the chiral recognition mechanism. Absolute configuration was reliably assigned by combining enantioselective HPLC with ECD spectroscopy, establishing that the (*R*)‐enantiomers of the series studied are consistently the more retained on the Chiralpak AD‐3 CSP. As previously reported, analytes with high enantioselectivity are especially valuable in mechanistic studies because they accentuate subtle differences in binding interactions. The chromatographic and spectroscopic data collected here may therefore provide a solid foundation for future computational studies aimed at elucidating the recognition mechanism of Chiralpak AD‐3 CSP.

## Author Contributions

**Selen Gözde Kaya**: investigation, data curation, writing – original draft, writing – review and editing. **Alessia Raucci**: investigation, data curation, visualization, writing – original draft, writing – review and editing. **Clemens Zwergel**: data curation, writing – original draft, writing – review and editing. **Antonello Mai**: resources, writing – original draft, writing – review and editing. acknowledgments. **Sergio Valente**: conceptualization, methodology, resources, writing – original draft, writing – review and editing, supervision, funding acquisition. **Roberto Cirilli**: conceptualization, methodology, investigation, resources, data curation, visualization, writing – original draft, writing – review and editing, supervision, funding acquisition.

## Conflicts of Interest

The authors declare no conflicts of interest.

## Supporting information




**Supporting File**: elps70109‐sup‐0001‐SuppMat.docx.

## Data Availability

The data are available from the corresponding authors upon reasonable request.
