# Supplementary material for: Probing Chiral Recognition on Amylose Tris(3,5‐Dimethylphenylcarbamate) Using Cinnamyl 2‐Aminoanilides: The Subtle Impact of Aliphatic Substituents
Source: Electrophoresis. 2026 May 19;47(7):613–9. doi: 10.1002/elps.70109 (PMC13378182; doi:10.1002/elps.70109)
Supplement: Supplementary file 1 — Supporting File: elps70109‐sup‐0001‐SuppMat.docx. [file ELPS-47--s001.docx]

**SUPPORTING MATERIALS**

**Probing Chiral Recognition on Amylose Tris(3,5-dimethylphenylcarbamate) Using cinnamyl 2-aminoanilides: The Subtle Impact of Aliphatic Substituents**

**Selen Gözde Kaya^a,b^, Alessia Raucci^a^, Clemens Zwergel^a^, Antonello Mai^a^, Sergio Valente****^a^**^^[[1]](#footnote-1)^*^**, Roberto Cirilli**^c^[[2]](#footnote-2)^*^

^a^ Dipartimento di Chimica e Tecnologie del Farmaco, Università degli Studi di Roma “La Sapienza” P.le A. Moro 5, 00185 Rome, Italy.

^b^ Gazi University, Faculty of Pharmacy, Department of Pharmaceutical Chemistry, Ankara, Türkiye

^c^ Centro Nazionale per il Controllo e la Valutazione dei Farmaci, Istituto Superiore di Sanità Viale Regina Elena 299, 00161 Rome, Italy

***Synthesis of 3-7***

The title compounds were prepared using an optimized procedure. Starting from the key intermediate, ethyl 4-aminocinnamate, the proper arylacetic acids were coupled with it in the presence of triethylamine and hexafluorophosphate azabenzotriazole tetramethyl uronium (HATU). This afforded the intermediate esters **8**-**13**, which were then hydrolyzed in a basic medium to give the related acrylic acids **14**-**19**. Treatment of **14-19** with benzotriazole-1-yloxytris(dimethylamino)phosphonium hexafluorophosphate (Bop-reagent), triethylamine, and 1,2-phenylendiamine or N-Methyl-1,2-phenylenediamine afforded the corresponding final compounds **1-7** (Scheme 1).

**Scheme 1.** Reagents and conditions: a) properly substituted arylacetic acid, Et_3_N, HATU reagent, dry DMF, N_2_, rt; b) LiOH, THF/H_2_O, rt; c) 1) Et_3_N, BOP reagent, dry DMF, N_2_; 2) 1,2-phenylendiamine, dry DMF, N_2_, rt.

***Chemistry***

Melting points were determined on a Buchi 530 melting point apparatus and are uncorrected. ^1^H NMR spectra were recorded at 400 MHz on a Bruker AC 400 spectrometer; chemical shifts are reported in δ (ppm) units relative to the internal standard tetramethylsilane (Me_4_Si). All compounds were routinely checked by TLC,^1^H-NMR. TLC was performed on aluminium-backed silica gel plates (Merck DC, Alufolien Kieselgel 60 F254) with spots visualised under UV light. All solvents were of reagent grade and, when necessary, were purified and dried by standard methods. Concentration of solutions after reactions was achieved using a rotary evaporator operating at a reduced pressure of approximately 20 Torr. Analytical results are within ±0.40% of the theoretical values. All chemicals were purchased from Sigma-Aldrich Chemistry (Milan, Italy), Flurochem (Manchester, UK), or BLDPharm (Hamburg, Germany) and were of the highest purity.

The chemical and physical properties of intermediates **8**,**9**, **11**, **15** and **17,** as well as final compounds **1**, **2** and **5,** correspond to those reported in.

The final compounds **1-7** possess>95% purity, as confirmed by ^1^H NMR and elemental analysis.

**General Procedure for the Synthesis of Ethyl 3-(4- Acylaminophenyl)-2-propenoates (8-13). Example: ethyl (E)-3-(4-(2-phenylhexanamido)phenyl)acrylate (12).**

Triethylamine (0.19 mmol, 0.26 mL) and HATU reagent (0.54 mmol, 0.205 g) were added under a nitrogen atmosphere to a solution of 2-phenylpentanoic acid (0.48 mmol, 0.086 g) in dry DMF (3 mL), followed by (E)-ethyl 3-(4-aminophenyl)acrylate (0.48 mmol, 0.092 g) and left stirring for 4 h at room temperature. The reaction mixture was then poured into water (30 mL), the organic layer was separated, and the aqueous layer was extracted with dichloromethane (3 × 30 mL). The combined organic layers were washed with brine (30 mL), dried over sodium sulphate and evaporated. The residue was chromatographed over silica gel, eluting with a 1:5 ethyl acetate/hexane mixture, to provide the desired compound. Mp: colourless oil; Yield: 58.6%; ^1^H NMR (CDCl_3_) δ 0.88-0.92 (d, 3H, CHCH_2_CH_2_CH_2_C*H_3_*), 1.21-1.42 (m, 7H, CHCH_2_C*H_2_*C*H_2_*CH_3_ and OCH_2_C*H_3_*),1.84-1.91 (m, 1H, CHC*H_2_*CH_2_CH_2_CH_3_), 2.24-2.31 (m, 1H, CHC*H_2_*CH_2_CH_2_CH_3_), 3.48-3.52 (t, 1H, PhC*H*CO), 4.24-4.30 (q, 2H, OC*H*_2_CH_3_), 6.34-6.38 (d, 1H, PhCH=C*H*COOEt), 7.13 (s, 1H, CON*H*Ph), 7.28-7.51 (m, 9H, benzene protons), 7.61-7.65 (d, 1H, PhC*H*=CHCOOEt) ppm.

ethyl (E)-3-(4-(2-phenylpropanamido)phenyl)acrylate (**8**): the chemical and physical data are according to the literature.

ethyl (E)-3-(4-(3-methyl-2-phenylbutanamido)phenyl)acrylate (**9**): the chemical and physical data are according to the literature.

ethyl (E)-3-(4-(2-phenylpentanamido)phenyl)acrylate (**10**): Mp: colourless oil; Yield: 76.0%; ^1^H NMR (CDCl_3_) δ 0.84-0.88 (d, 3H, CHCH_2_CH_2_C*H_3_*), 1.15-1.25 (m, 5H, CHCH_2_C*H_2_*CH_3_ and OCH_2_C*H_3_*),1.72-1.81 (m, 1H, CHC*H_2_*CH_2_CH_3_), 2.10-2.19 (m, 1H, CHC*H_2_*CH_2_CH_3_), 3.41-3.45 (t, 1H, PhC*H*CO), 4.15-4.20 (q, 2H, OC*H*_2_CH_3_), 6.25-6.29 (d, 1H, PhCH=C*H*COOEt), 7.07 (s, 1H, CON*H*Ph), 7.20-7.39 (m, 9H, benzene protons), 7.51-7.55 (d, 1H, PhC*H*=CHCOOEt) ppm.

ethyl (E)-3-(4-(2-phenylpent-4-enamido)phenyl)acrylate (**11**): the chemical and physical data are according to the literature.

ethyl (E)-3-(4-(5-methyl-2-phenylhex-4-enamido)phenyl)acrylate (**13**): Mp: colourless oil; Yield: 61.7%; ^1^H NMR (CDCl_3_) δ 1.29-1.35 (t, 3H, OCH_2_C*H_3_*), 1.60 (s, 3H, CHCH_2_CH=C(C*H_3_*)_2_), 1.67 (s, 3H, CHCH_2_CH=C(C*H_3_*)_2_), 2.55-2.57 (s, 1H, CHC*H_2_*CH=C(CH_3_)_2_, 2.92-2.95 (s, 1H, CHC*H_2_*CH=C(CH_3_)_2_, 3.50-3.54 (t, 1H, PhC*H*CO), 4.24-4.28 (q, 2H, OC*H_2_*CH_3_), 5.10 (s, 1H, CHCH_2_C*H*=C(CH_3_)_2_), 6.34-6.38 (d, 1H, PhCH=C*H*COOEt), 7.15 (s, 1H, CON*H*Ph), 7.28-7.50 (m, 9H, benzene protons), 7.61-7.65 (d, 1H, PhC*H*=CHCOOEt) ppm.

**General Procedure for the Synthesis of 3-(4*-*Acylaminophenyl)-2-propenoic Acids (14-19). Example: (E)-3-(4-(2-phenylhexanamido)phenyl)acrylic acid (18).**

A mixture of **12** (0.32 mmol, 0.11 g) and lithium hydroxide hydrate (0.65 mmol, 0.027 g) in tetrahydrofuran/water (3 mL/3 mL) was stirred at room temperature. After 24 h, 2N HCl was added to the mixture until the pH reached 5, and the resulting solid was filtered and recrystallised from acetonitrile/methanol to give pure compound **18**. Mp: 167-169°C; colourless solid; Yield: 99.0%; recryst solvent: benzene/acetonitrile; ^1^H NMR (DMSO-*d*6) δ 0.83-0.87 (d, 3H, CHCH_2_CH_2_CH_2_C*H_3_*), 1.17-1.35 (m, 4H, CHCH_2_C*H_2_* C*H_2_*CH_3_), 1.66-1.71 (m, 1H, CHC*H_2_*CH_2_ CH_2_CH_3_), 2.00-2.09 (m, 1H, CHC*H_2_*CH_2_ CH_2_CH_3_), 3.65-3.69 (t, 1H, PhC*H*CO),6.38-6.42 (d, 1H, PhCH=C*H*COOH), 7.23-7.26 (t, 1H, benzene proton), 7.31-7.35 (t, 2H, benzene protons), 7.39-7.40 (d, 2H, benzene protons), 7.46-7.50 (d, 1H, PhC*H*=CHCOOH), 7.55-7.65 (m, 4H, benzene protons), 10.28 (bs, 1H, CON*H*Ph), 12.35 (bs, 1H, COOH) ppm.

(E)-3-(4-(2-phenylpropanamido)phenyl)acrylic acid (**14**): the chemical and physical data are according to the literature.

(E)-3-(4-(3-methyl-2-phenylbutanamido)phenyl)acrylic acid (**15**): the chemical and physical data are according to the literature.

(E)-3-(4-(2-phenylpentanamido)phenyl)acrylic acid (**16**): Mp: 153-155°C; colourless solid; Yield: 88.5%; recryst solvent: benzene/acetonitrile; ^1^H NMR (DMSO-*d*6) δ 0.89-0.92 (d, 3H, CHCH_2_CH_2_C*H_3_*), 1.24-1.36 (m, 2H, CHCH_2_C*H_2_*CH_3_), 1.63-1.71 (m, 1H, CHC*H_2_*CH_2_CH_3_), 1.99-2.08 (m, 1H, CHC*H_2_*CH_2_CH_3_), 3.68-3.72 (t, 1H, PhC*H*CO), 6.38-6.42 (d, 1H, PhCH=C*H*COOEt), 7.22-7.40 (m, 5H, benzene protons), 7.49-7.53 (d, 1H, PhC*H*=CHCOOEt), 7.60-7.66 (m, 4H, benzene protons), 10.29 (bs, 1H, CON*H*Ph), 12.27 (bs, 1H, COOH) ppm.

(E)-3-(4-(2-phenylpent-4-enamido)phenyl)acrylic acid(**17**): the chemical and physical data are according to the literature.

(E)-3-(4-(5-methyl-2-phenylhex-4-enamido)phenyl)acrylic acid (**19**): Mp: 149-151°C; colourless solid; Yield: 93.5%; recryst solvent: benzene/acetonitrile; ^1^H NMR (DMSO-*d*6) δ 1.58 (s, 3H, CHCH_2_CH=C(C*H_3_*)_2_), 1.61 (s, 3H, CHCH_2_CH=C(C*H_3_*)_2_), 2.35-2.39 (s, 1H, CHC*H_2_*CH=C(CH_3_)_2_, 2.72-2.76 (s, 1H, CHC*H_2_*CH=C(CH_3_)_2_, 3.66-3.69 (t, 1H, PhC*H*CO), 5.05 (s, 1H, CHCH_2_C*H*=C(CH_3_)_2_), 6.37-6.41 (d, 1H, PhCH=C*H*COOH), 7.23-7.26 (t, 1H, benzene proton), 7.31-7.35 (t, 2H, benzene protons), 7.39-7.41 (d, 2H, benzene protons), 7.46-7.64 (m, 4H, PhC*H*=CHCOOH and benzene protons), 10.25 (bs, 1H, CON*H*Ph), 12.29 (bs, 1H, COOH) ppm.

**General Procedure for the Synthesis of the final compounds (1-7). Example: (E)-N-(4-(3-((2-aminophenyl)amino)-3-oxoprop-1-en-1-yl)phenyl)-5-methyl-2-phenylhex-4-enamide (7).**

Triethylamine (0.95 mmol, 0.13 mL) and BOP reagent (0.28 mmol, 0.127 g) were added under a nitrogen atmosphere to a solution of compound **19** (0.24 mmol, 0.105 g) in dry DMF (3 mL), and the resulting mixture was stirred for 30 min. After this time, 1,2-phenylendiamine (0.24 mmol, 0.026 g) was added, and the stirring was continued for a further 4 h. The reaction was quenched by water (15 mL), and the precipitate was filtered, washed with water (3 × 10 mL) and dried. The solid residue was chromatographed over silica gel eluting with chloroform/isopropanol/ammonia 30:1:01 ratio to provide the desired compound **7**, which was recrystallised by acetonitrile/methanol. Mp: 197-199°C; white solid; Yield: 38.5%; recryst solvent: acetonitrile/methanol; ^1^H NMR (DMSO-*d*6) δ 1.59 (s, 3H, CHCH_2_CH=C(C*H_3_*)_2_), 1.62 (s, 3H, CHCH_2_CH=C(C*H_3_*)_2_), 2.35-2.40 (s, 1H, CHC*H_2_*CH=C(CH_3_)_2_, 2.75-2.79 (s, 1H, CHC*H_2_*CH=C(CH_3_)_2_, 3.67-3.70 (t, 1H, PhC*H*CO), 4.94 (bs, 2H, N*H_2_*), 5.07 (s, 1H, CHCH_2_C*H*=C(CH_3_)_2_), 6.56-6.60 (m, 1H, aniline proton), 6.76-6.81 (m, 2H, PhCH=C*H*COOEt and aniline proton), 6.90-6.94 (m, 1H, aniline proton), 7.23-7.27 (m, 1H, benzene protons), 7.32-7.37 (m, 3H, aniline and benzene protons), 7.40-7.42 (d, 2H, benzene protons), 7.45-7.49 (d, 1H, PhC*H*=CHCOOEt), 7.54-7.56 (d, 2H, benzene protons), 7.65-7.68 (d, 2H, benzene protons), 9.33 (bs, 1H, CON*H*aniline), 10.26 (bs, 1H, CON*H*Ph), ppm.

(E)-N-(2-aminophenyl)-3-(4-(2-phenylpropanamido)phenyl)acrylamide (**1**): the chemical and physical data are according to the literature.

(E)-N-(4-(3-((2-aminophenyl)amino)-3-oxoprop-1-en-1-yl)phenyl)-3-methyl-2-phenylbutanamide (**2**): the chemical and physical data are according to the literature.

(E)-N-(4-(3-((2-aminophenyl)amino)-3-oxoprop-1-en-1-yl)phenyl)-2-phenylpentanamide (**3**): Mp: 225-227°C; yellowish solid; Yield: 28.1%; recryst solvent: benzene/acetonitrile; ^1^H NMR (DMSO-*d*6) δ 0.87-0.93 (d, 3H, CHCH_2_CH_2_C*H_3_*), 1.20-1.39 (m, 2H, CHCH_2_C*H_2_*CH_3_), 1.63-1.72 (m, 1H, CHC*H_2_*CH_2_CH_3_), 2.00-2.09 (m, 1H, CHC*H_2_*CH_2_CH_3_), 3.69-3.73 (t, 1H, PhC*H*CO), 4.94 (bs, 2H, N*H_2_*), 6.56-6.60 (m, 1H, aniline proton), 6.74-6.81 (m, 2H, PhCH=C*H*COOEt and aniline proton), 6.89-6.93 (m, 1H, aniline proton), 7.23-7.27 (m, 1H, benzene protons), 7.32-7.34 (m, 3H, aniline and benzene protons), 7.36-7.40 (d, 2H, benzene protons), 7.46-7.49 (d, 1H, PhC*H*=CHCOOEt), 7.54-7.57 (d, 2H, benzene protons), 7.66-7.68 (d, 2H, benzene protons), 9.33 (bs, 1H, CON*H*aniline), 10.29 (bs, 1H, CON*H*Ph), ppm.

(E)-N-(4-(3-((2-(methylamino)phenyl)amino)-3-oxoprop-1-en-1-yl)phenyl)-2-phenylpentanamide (**4**): Mp: 213-215°C; yellowish solid; Yield: 27.2%; recryst solvent:acetonitrile/methanol; ^1^H NMR (DMSO-*d*6) δ 0.90-0.93 (d, 3H, CHCH_2_CH_2_C*H_3_*), 1.20-1.29 (m, 2H, CHCH_2_C*H_2_*CH_3_), 1.63-1.70 (m, 1H, CHC*H_2_*CH_2_CH_3_), 2.00-2.07 (m, 1H, CHC*H_2_*CH_2_CH_3_),2.72.2.75 (d, 3H, NHC*H_3_*), 3.69-3.73 (t, 1H, PhC*H*CO), 5.13-5.14 (bs, 2H, N*H*CH_3_), 6.59-6.63 (t, 2H, aniline proton), 6.76-6.80 (m, 1H, PhCH=C*H*COOEt), 7.04-7.08 (t, 1H, aniline proton), 7.23-7.34 (m, 4H, aniline and benzene protons), 7.36-7.40 (d, 2H, benzene protons), 7.44-7.48 (d, 1H, PhC*H*=CHCOOEt), 7.53-7.55 (d, 2H, benzene protons), 7.66-7.68 (d, 2H, benzene protons), 9.28 (bs, 1H, CON*H*aniline), 10.29 (bs, 1H, CON*H*Ph), ppm.

(E)-N-(4-(3-((2-aminophenyl)amino)-3-oxoprop-1-en-1-yl)phenyl)-2-phenylpent-4-enamide (**5**): the chemical and physical data are according to the literature.

(E)-N-(4-(3-((2-aminophenyl)amino)-3-oxoprop-1-en-1-yl)phenyl)-2-phenylhexanamide (6): Mp: 200-202°C; yellowish solid; Yield: 35.5%; recryst solvent: acetonitrile/methanol; ^1^H NMR (DMSO-*d*6) δ0.84-0.88 (d, 3H, CH_2_CH_2_CH_2_C*H_3_*), 1.20-1.36 (m, 4H, CHCH_2_C*H_2_* C*H_2_*CH_3_), 1.69-1.74 (m, 1H, CHC*H_2_*CH_2_ CH_2_CH_3_), 2.00-2.08 (m, 1H, CHC*H_2_*CH_2_ CH_2_CH_3_), 3.66-3.70 (t, 1H, PhC*H*CO), 4.94 (bs, 2H, N*H_2_*), 6.56-6.60 (m, 1H, aniline proton), 6.74-6.81 (m, 2H, PhCH=C*H*COOEt and aniline proton), 6.89-6.93 (m, 1H, aniline proton), 7.23-7.26 (m, 1H, benzene protons), 7.33-7.35 (m, 3H, aniline and benzene protons), 7.39-7.41 (d, 2H, benzene protons), 7.41-7.45 (d, 1H, PhC*H*=CHCOOEt), 7.54-7.56 (d, 2H, benzene protons), 7.66-7.68 (d, 2H, benzene protons), 9.33 (bs, 1H, CON*H*aniline), 10.27 (bs, 1H, CON*H*Ph), ppm.

1. *Corresponding author. E-mail address: roberto.cirilli@iss.it (Dr. Roberto Cirilli) [↑](#footnote-ref-1)
2. *Corresponding author. E-mail address: sergio.valente@uniroma1.it (Prof. Sergio Valente) [↑](#footnote-ref-2)
